# Beyond the Metabolic Syndrome: Non-Obvious Complications of Obesity in Children

**DOI:** 10.3390/children10121905

**Published:** 2023-12-09

**Authors:** Agnieszka Kozioł-Kozakowska, Dominika Januś, Anna Stępniewska, Ewa Szczudlik, Anna Stochel-Gaudyn, Małgorzata Wójcik

**Affiliations:** 1 Department of Pediatrics, Gastroenterology and Nutrition, Institute of Pediatrics, Jagiellonian University Medical College, 31-008 Cracow, Poland; agnieszka.koziol-kozakowska@uj.edu.pl (A.K.-K.); a.stochel-gaudyn@j.edu.pl (A.S.-G.); 2 Interclinical Center for the Treatment of Childhood Obesity, University Children’s Hospital of Krakow, 30-663 Kraków, Poland; anna.stepniewska@uj.edu.pl (A.S.); e.szczudlik@uj.edu.pl (E.S.); 3 Department of Pediatric and Adolescent Endocrinology, Pediatric Institute, Jagiellonian University Medical College, 31-008 Kraków, Poland; dominika.janus@uj.edu.pl

**Keywords:** obesity, children, adolescents, obesity complications

## Abstract

Obesity is currently one of the most significant public health challenges worldwide due to the continuous increase in obesity rates among children, especially younger children. Complications related to obesity, including serious ones, are increasingly being diagnosed in younger children. A search was performed from January 2023 to September 2023 using the PubMed, Cochrane Library, Science Direct, MEDLINE, and EBSCO databases. The focus was on English-language meta-analyses, systematic reviews, randomized clinical trials, and observational studies worldwide. Four main topics were defined as follows: disorders of glucose metabolism; liver disease associated with childhood obesity; the relationship between respiratory disorders and obesity in children; and the effects of obesity on the hypothalamic–pituitary–gonadal axis and puberty. Understanding potential complications and their underlying mechanisms can expedite the diagnostic process and enhance the effectiveness of treatment. We aspire that this study will bring insight into the often-overlooked complications associated with obesity.

## 1. Introduction

Obesity is currently a prevalent health issue and one of the foremost public health challenges worldwide. In the last four decades, there has been a rise in the prevalence of childhood obesity among children aged 5 to 19 years in most regions and countries. In 2016, it was determined that the number of obese young children (<5 years old) globally exceeded 41 million [1]. Additionally, there has been a noticeable increase in the occurrence of severe obesity (morbid, extreme obesity) in progressively younger age groups.

It is well established that excess body weight in adults is linked to comorbid cardiometabolic and psychosocial diseases as well as premature mortality. Complications of obesity, leading to at least 2.6 million annual deaths, are increasingly diagnosed in individuals under 18 years of age [2].

The global surge in childhood obesity can be attributed to various factors. Firstly, there has been a widespread shift in the diets of young people. Scientific evidence unequivocally confirms a significant increase in both the size and frequency of meals consumed by children and adolescents over the last 40 years. There is also a notable rise in the consumption of high-energy, processed foods that are rich in fats and sugars but deficient in vitamins, minerals, and other essential microelements. Simultaneously, lifestyle changes resulting from progressive urbanization have compelled a reduction in physical activity.

Equally important is the exposure of children and adolescents to chronic stress stimuli, inadequate stress coping mechanisms, a lack of support, and disruptions in their length and quality of sleep. These factors indirectly lead to the development of compensatory mechanisms, often associated with the consumption of excess high-calorie foods and drinks, even at night. All these environmental factors strongly interact with genetic predisposition, which significantly influences body weight control [3]. Obesity, being a multifaceted health concern, necessitates a nuanced understanding of its diverse complications, both obvious and non-obvious. Non-obvious complications refer to health issues or consequences that may not be immediately apparent or visible, especially in the early stages of obesity. These complications may not present obvious physical symptoms or signs, making their detection and diagnosis more challenging. Non-obvious complications may involve subtle or internal changes that can have significant impacts on health over time. Identifying and understanding these non-obvious complications is crucial for comprehensive healthcare, as it enables early intervention and targeted management to mitigate potential risks associated with obesity. The study’s objective is to identify and discuss non-obvious complications of obesity in children, contributing to a better understanding of the mechanisms accompanying childhood obesity.

## 2. Methods

**Figure 1:**
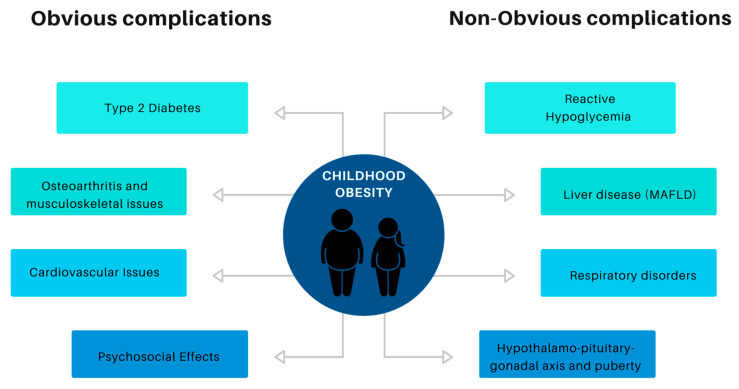
Classification of complications of childhood obesity.

A search using the keywords “obvious” and “non-obvious” obesity complications was conducted across the PubMed, Cochrane Library, Science Direct, and EBSCO databases from January 2023 to September 2023. The objective was to identify worldwide English-language meta-analyses, systematic reviews, randomized clinical trials, and observational studies. As a result, the complications identified in children are shown in Figure 1. Ultimately, the following 4 main topics were identified: disorders of glucose metabolism; liver disease related to childhood obesity; the relationship between respiratory disorders and obesity in children; and the effects of obesity on the hypothalamic–pituitary–gonadal axis and puberty.

## 3. Disorders of Glucose Metabolism

Overt and persistent hyperglycemia in obese children is not as common as in adults [4]. Type 2 diabetes occurs in only 1–2% of obese pediatric patients, with the highest prevalence only in genetically predisposed ethnic groups, for example, Native American, Canadian First Nation, Indigenous Australian, African American, Hispanic, etc. This does not mean, however, that the pediatric population affected by obesity is free from glucose metabolism disorders [5]. However, they may be less obvious, such as reactive hypoglycemia. The term “reactive hypoglycemia” was first used in 1924 by Harris, who used this name to describe a set of symptoms similar to those occurring in diabetic patients after insulin administration [6]. Symptoms such as feelings of hunger and behavioral and mood disorders, from agitation and irritation to slowness and drowsiness, weakness, even loss of consciousness, increased sweating, and heart palpitations, occur in people without diabetes and are associated with a rapid decrease in blood glucose concentration in response to a carbohydrate-rich food stimulus. Symptoms disappear after consuming simple carbohydrates. Their direct cause is the incorrect secretion of insulin at a time and in an amount that is not adapted to the body’s current needs. Recent studies indicate that individuals with obesity may have blood glucose levels below 70 mg/dL without significant hypoglycemic symptoms, except for hunger [7]. That, in turn, may be responsible for snacking behavior [8]. The cause of reactive hypoglycemia is inappropriate insulin secretion. In children with obesity and reactive hypoglycemia, the first phase of insulin secretion is altered in response to the carbohydrates supplied. Subsequently, a large amount of insulin is secreted as a compensatory factor, and, as a consequence, serum glucose concentration is excessively reduced 3–5 h after a meal. As shown in studies using continuous glucose monitoring, reactive hypoglycemia may be experienced by up to 50% of obese patients during an oral glucose tolerance test (OGTT) [7]. Interestingly, only a few report the symptoms described above. In the rest, the disorder is asymptomatic. Traditional glucose measurements involving venipuncture or finger pricks fail to capture the exact fluctuations in blood glucose over time, specifically the variability in glucose levels. Disorders can only be documented using continuous glucose monitoring systems. Alternatively, when interpreting OGTT results, it is worth paying attention not only to the high glycemia values at 120 min after a load but also to those that are within the reference range but are lower than the preload glycemia values. In obese patients with symptoms typical of reactive hypoglycemia or an uncontrollable tendency to snack on sweet snacks, it is worth considering a prolonged oral glucose tolerance test (up to 180 or 240 min) [8]. The basis of treatment is to modify the diet and make the patient aware of the importance of eating regular, well-composed meals and avoiding carbohydrates in the diet. In some situations, metformin or GLP-1 analogues may be helpful. That, however, needs further investigation in the pediatric population.

## 4. Liver Disease Related to Childhood Obesity

One of the many complications related to childhood obesity is pediatric fatty liver disease associated with metabolic dysfunction (MAFLD), previously known as non-alcoholic liver disease (NAFLD). The diagnostic criteria include liver histology (biopsy), imaging (ultrasound, MRI), or blood sampling (evidence of intrahepatic fat accumulation). It is crucial to underline that the relatively new name MAFLD emphasizes the importance of metabolic causes of the disease, which were not previously included in NAFLD.

In the last two decades, the prevalence of this disease has doubled, and estimates for the European pediatric population vary from 13% to 25% among children aged 3–18 years, depending on the diagnostic criteria. [9,10]. Among obese children in the USA, approximately 38% are estimated to have fatty liver disease [11]. In a Polish retrospective study, steatosis was reported in 4.2% of children aged 6 months to 18 years, with 55.6% of these children being overweight [12].

The manifestations of MAFLD may include steatosis alone, steatohepatitis with and without fibrosis, or even cirrhosis. Since a definitive diagnosis requires a liver biopsy, which is an invasive procedure, the disease is often suspected based on biochemical results (elevated aspartate aminotransferase (AST) or alanine aminotransferase (ALT) concentrations) or imaging, particularly a liver ultrasound, including elastography. A study conducted among adults in the USA using ultrasound elastography showed that 24% of participants had steatosis and 4.4% had significant fibrosis [13]. A corresponding study among obese children revealed that both AST and ALT were significantly higher than in the norm-weight control group. Additionally, 25% of participants showed steatosis on an ultrasound, and elastography showed higher values reflecting fibrosis in the obese pediatric population [14]. The long-term results of MAFLD are not completely defined, as much of the data, especially among the pediatric population, come from retrospective studies and case reports [15,16]. Nevertheless, it is important to underline that, although rare, severe complications such as cirrhosis, liver failure, and hepatocarcinomas have been reported [17]. Obesity in late adolescence and early adulthood appears to increase the risk of severe MAFLD complications, such as liver cancer [18]. The duration of the disease may also play an important role. Two randomized controlled trials among pediatric patients with fatty liver disease showed disease progression in a two-year span, based on histological examinations. Disease progression was also correlated with insulin resistance [19]. According to studies focusing on children who underwent liver biopsy due to fatty liver disease, up to 70% of them showed some degree of fibrosis by the time of diagnosis, including advanced fibrosis in 16–30% of cases. End-stage liver failure requiring liver transplantation due to fatty liver disease was reported even in very young children. According to the USA transplantation registry, from 1987 to 2012, 14 children younger than 18 underwent this procedure [20]. The severity of MAFLD appears to be linked to both the age of the patient and the stage of the disease. It is also twice as common in adolescents with type 2 diabetes [21]. Nevertheless, when treating an obese pediatric patient with fatty liver disease, it is crucial to remember to look for other treatable causes of liver dysfunction, especially if there is poor improvement after weight loss, such as Wilson’s disease, viral hepatitis, or autoimmune hepatitis.

## 5. Relationship between Respiratory Disorders and Obesity in Children

Asthma is a prevalent chronic disease among children, with a prevalence varying between 1% and 18% [22]. The recently observed rise in the occurrence of obesity and asthma in the pediatric group and their interrelation has been the subject of numerous studies [23]. However, this connection is not well understood [24,25,26]. We often encounter a vicious cycle where asthma contributes to obesity and vice versa. In children with inadequately managed asthma, multiple factors are typically at play, including the utilization of systemic corticosteroids or decreased physical activity. These factors can ultimately disrupt carbohydrate metabolism and elevate the risk of obesity.

Recent findings have indicated a potential connection between the development of asthma and gradual weight gain over time [23,27,28,29]. According to Lang et al., school-aged children before reaching puberty are most susceptible to developing asthma linked to obesity, specifically those between the ages of 7 and 11 [30]. Their suggestion was that the initiation of asthma could be influenced by both the duration and severity of being overweight, and they proposed that the period before puberty, especially among girls, might be a particularly high-risk timeframe for the development of asthma associated with obesity. In their study, Chen et al. monitored the emergence of obesity over a 10-year follow-up in children with asthma. They noted that children who were not obese initially had a 51% greater likelihood of developing obesity when compared to children who did not have a prior asthma diagnosis. Nonetheless, the presence of obesity was not directly linked to the future development of asthma [30,31,32].

Asthma presents with clinically diverse symptoms, such as wheezing, shortness of breath, chest tightness, and cough, all originating from chronic airway inflammation. Obesity has been associated with a decline in lung function, as both forced expiratory volume in one second (FEV1) and forced vital capacity (FVC) exhibit a negative correlation with waist circumference [33,34,35]. On average, every 1 cm increase in waist circumference was linked to a 13 mL decrease in FEV1 and an 11 mL decrease in FVC [36].

The “Obese-Asthma” phenotype displays a distinct molecular pattern compared to the classic form. The Th2-low pattern is characterized by a predominant presence of neutrophils in the bronchial mucosa, alongside low levels of IgE and limited eosinophilic infiltration [37,38]. Excessive adipose tissue accumulation has proinflammatory effects associated with increased leptin production, hypoxia, focal adipocyte necrosis, and activation of neutrophils, reactive oxygen, macrophages, and natural killer cells [39]. Additionally, obesity-related insulin resistance generates proinflammatory molecules, promoting the development of inflammation [29,40]. Furthermore, coexisting hyperinsulinemia, by inhibiting the presynaptic processes of M2 muscarinic receptors, leads to bronchial hyperreactivity. In the research conducted by Leija-Martínez et al., the presence of “Obese-asthma” alongside an elevated Th17 immune response was correlated with airway hyperreactivity (AHR), severe asthma, and resistance to corticosteroid treatment [29,40,41,42].

In the study by Machado et al., it was found that leptin may be a potential predictor of asthma control in children. A weak predictive value was shown for BMI and adiponectin [43]. 

Elevated levels of CRP, often observed in obese patients due to cytokine stimulation from adipose tissues, have been correlated with the risk of asthma development and its severity [35]. Excessive abdominal tissue deposition also exerts a mechanical influence on the respiratory system by reducing chest expansion and decreasing tidal volume and residual capacity [44]. Afshar-Mohajer et al.’s research revealed that obese children with asthma are more susceptible to the effects of air pollution, specifically PM 2.5, compared to non-obese children. This increased susceptibility was attributed to their higher tidal volumes and minute ventilation. These factors contribute to an elevated risk of experiencing more severe and uncontrolled asthma [45].

The primary approach to addressing pediatric obesity involves lifestyle modifications, including moderate energy consumption, increased physical activity, decreased sedentary behaviors, and active family engagement in the treatment process. Weight reduction in asthmatic children not only decreases the clinical symptoms of asthma but also improves lung function and asthma control, leading to a better quality of life [46]. It has been reported that even a 5–10% reduction in weight can result in improved asthma outcomes [47]. Willeboardse et al. conducted a study monitoring 87 children who were both asthmatic and overweight or obese over 18 months [46]. The intervention involved a combination of activities, including sports sessions, dietary adjustments, parental participation, counseling, and behavioral therapy. Over the course of the study, clinically significant improvements in body weight, lung function, and asthma-related characteristics were observed in both the intervention and control groups. However, certain effects were more prominent in the intervention group, such as FVC, asthma control, and overall quality of life. This suggests that weight-reduction interventions can have clinical benefits for children with asthma.

It is important to note that steroids are less effective for individuals with asthma who are obese compared to those with a lower BMI [48]. Specifically, obese individuals, particularly those with severe obesity, have a reduced likelihood of achieving asthma control. While there have been conflicting reports on this matter, a retrospective cohort study involving data from six European centers found that obesity was not considered a significant risk factor for asthma exacerbations [49]. Up to 40% of children and adolescents with obesity may experience obesity hypoventilation syndrome (OHS) [50]. It typically manifests during sleep through recurrent episodes of either shallow breathing or complete cessation of airflow through the upper airways while chest and abdominal movements continue. Signs of OHS may encompass mouth breathing, disruptions in breathing rhythms, nighttime snoring, difficulties with concentration, as well as restlessness, headaches, and excessive daytime drowsiness. Ventilation dysfunction leads to multiple episodes of hypoxia and hypercapnia, negatively affecting the functioning of the entire body, including growth, development, and psycho-emotional well-being, and leading to the development of other complications of obesity, such as hypertension [51]. Several studies have explored the connection between body mass index measurements and lung function parameters, along with polysomnographic evaluations, in children with asthma. In a retrospective study that examined the polysomnographic data of 448 children aged 7 to 18 years with asthma, efforts were made to investigate the relationship between spirometry results, body mass index, and polysomnography parameters. This analysis also considered the influence of the medications administered. The study revealed that obese children had less favorable sleep patterns and more pronounced disruptions in gas exchange compared to children of normal weight. Additionally, it was found that asthma medications, such as inhaled glucocorticosteroids and leukotriene antagonists, had an impact on sleep pattern disturbances, consequently triggering pathological activations of the sympathetic nervous system. Recognizing the presence of “obesity-related asthma” is vital to initiating personalized interventions for children. These interventions aim to encourage physical activity, cultivate healthy habits, and improve asthma self-care, ultimately reducing long-term morbidity and mortality.

## 6. Effects of Obesity on the Hypothalamic–Pituitary–Gonadal Axis and Puberty

### 6.1. Obese Girls

The increasing number of obese children is associated with a broad spectrum of gynecological consequences for adolescent girls, impacting them during adolescence and later in adulthood. Precocious puberty may have adverse effects on girls’ mental and psychosocial health. Obesity heightens the risk of low self-esteem and depression, particularly in girls who are prone to engaging in risky sexual conduct and demonstrate ineffective use of contraception. Irregular menses, amenorrhea, abnormal uterine bleeding, dysmenorrhea, and polycystic ovary syndrome (PCOS) at heightened rates may contribute to infertility, pregnancy complications, as well as breast and endometrial cancers later in adult life [52,53,54,55,56,57,58].

#### 6.1.1. Premature Puberty

In the 1970s, it was hypothesized that a “critical body weight” is crucial to starting the process of puberty [59]. Excessive levels of body fat are connected with an earlier onset of menarche [60]. Leptin, a hormone secreted by adipocytes, provides information on an organism’s nutritional status and acts via the stimulation of Kiss-1 neurons as a factor in accelerating puberty. Levels of leptin and kisspeptin are higher in obese girls compared to healthy-weight girls [61].

In obese girls, the most common form of premature puberty is the early appearance of pubic and axillary hair (at <8 years of age). The excess of insulin, which is often observed in obese children, appears to stimulate androgen secretion in ovarian theca cells, even in the absence of luteinizing hormone (LH), and promote excessive androgen production by the adrenal glands. Moreover, hyperinsulinemia decreases the hepatic excretion of sex hormone-binding globulin (SHBG), which consequently leads to increased androgen bioavailability (i.e., increased free testosterone) [62]. It may clinically present as the premature appearance of pubic and axillary hair and may be accompanied by the characteristic smell of sweat, which is typical of adolescence, mild acne, moderately accelerated growth and bone age, and isolated mild elevations in dehydroepiandrosterone sulfate (DHEAS) levels. Generally, the premature isolated development of pubic and/or axillary hair is considered a benign process that does not disturb the normal timing and course of puberty and does not affect the body’s final height. However, a connection was noted between the occurrence of adrenarche praecox and obesity and the risk of developing polycystic ovary syndrome in girls [63,64].

Another mechanism for the early onset of menstruation and puberty is increased aromatase activity in fat cells, which leads to higher estrogen levels and may lead to isolated thelarche—the less common manifestation of precocious puberty. Unlike in real central puberty, in this mild variant, levels of LH remain unchanged but may occur with mild increases in follicle-stimulating hormone (FSH). Growth rate and bone age do not accelerate. The mild forms of precocious puberty in obese children do not require treatment; they exhibit a stable or very slow progression.

#### 6.1.2. Menstrual Disorders, Polycystic Ovary Syndrome (PCOS), and Other Syndromes Connected with Obesity in Adolescent Girls

The menstrual cycle results from a complex balance and interaction of hormones. Any disruption to these mechanisms can impact its physiology. Obesity in pubertal girls and young women may be a contributing factor to ovarian dysfunction. The most common noticeable symptom of this dysfunction is an irregular menstrual cycle, which occurs twice as often as in slim girls of the same age. Additionally, obesity is also associated with a higher risk of other menstrual disorders, such as heavy or light bleeding, dysmenorrhea, oligomenorrhea, secondary amenorrhea, and polycystic ovary syndrome (PCOS). All of those disorders are related to disruptions in the hormonal balance between estrogens, gestagens, and androgens, which occur secondary to other hormonal disorders, mainly hyperleptinemia, hyperinsulinemia, and insulin resistance [62].

One of the most serious ovarian dysfunctions affected by obesity, with much more far-reaching general health consequences, is polycystic ovary syndrome (PCOS). It can manifest with a variety of clinical irregularities, including menstrual irregularities, acne, hirsutism, acanthosis nigricans, and seborrhea. Diagnosing it in some patients can be challenging during adolescence. In adolescent girls, it presents as irregular menstruation and clinical hyperandrogenism. Ovulation disorders that occur during the course of the syndrome may be a cause of fertility issues. The pathogenesis of PCOS is based on the disturbance of insulin secretion and its activity, and it is associated with potentially harmful health abnormalities such as hypertension, dyslipidemia, impaired glucose metabolism, obstructive sleep apnea, and non-alcoholic fatty liver disease. Moreover, it can lead to infertility and cardiovascular disease in adulthood [65]. Hyperinsulinemia is responsible for the increased secretion of androgens by the ovaries and adrenal glands, along with the reduced synthesis of sex hormone-binding globulin (SHBG), which consequently leads to excess androgens. According to the consensus of 2017 and 2018, PCOS can be diagnosed in adolescents if both of the following criteria are met: (1) the occurrence of menstrual disorders (including irregular menses, oligomenorrhea, and secondary amenorrhea) and (2) hyperandrogenism [66,67]. A diagnosis of PCOS can be made if at least 2 years have passed since the first menstruation and persistent disorders in menstruation persist for over 2 years. Other potential causes of menstrual irregularities and hyperandrogenism should be ruled out, including hypothyroidism, hypercortisolemia, hyperprolactinemia, congenital adrenal hyperplasia, and androgen-secreting tumors. According to the evidence-based recommendation from 2023, it is recommended not to diagnose PCOS during adolescence due to the overlap between the characteristics of physiological maturation and polycystic ovary syndrome. For adolescents displaying PCOS characteristics but not meeting diagnostic criteria, an “elevated risk” could be contemplated, and re-evaluation is advised at or before full reproductive maturity, typically 8 years after menarche. This includes those with PCOS characteristics before the start of the combined oral contraceptive pill (COCP), those with persisting characteristics, and those with significant weight gain in adolescence [68]. The goal of treatment is to restore normal monthly cycles. Weight loss is a necessary condition for a lasting treatment effect, but, most often, it is not sufficient at the beginning; therefore, it is usually necessary to use a contraceptive pill containing progestogen for an antiandrogenic effect. In very young patients or with contraindications to the administration of estrogens (venous thrombosis, migraine with aura), treatment with natural progesterone in the second phase of the cycle can be considered. For girls with PCOS and disorders of glucose metabolism or features of MAFLD, metformin may be recommended, which not only improves metabolic parameters but also has a positive effect on the regularity of cycles, monthly periods, and ovulation [66,67,68]. Metformin can ameliorate ovulation and the metabolic risk of PCOS, and, currently, together with hormonal contraceptives, it constitutes the basis of therapy in adolescents with PCOS. In addition to promoting weight loss and systemic metabolic effects, metformin has been proposed to exert influence at the ovarian level. Apart from its metabolic actions in the ovary, metformin has demonstrated the inhibition of in vitro androgen production in isolated human ovarian granulosa cells, with this impact being particularly notable in the presence of insulin. Furthermore, the androgen-lowering effects of metformin were found to be secondary to decreased circulating insulin levels and the subsequent reduced activity of steroidogenic enzymes. Studies have also proposed a significant role for ovarian AMP-dependent kinase in mediating the effects of metformin.

### 6.2. Obese Boys

#### 6.2.1. Gynecomastia (Gm), Pubertal Gynecomastia (pGm), and Pseudogynecomastia (Pseudo-Gm) in Adolescent Males

Gynecomastia (Gm) is one of the most embarrassing and hidden problems in overweight/obese adolescent boys. Gm is usually physiological during puberty but might also be pathological [69,70]. Physiological pubertal gynecomastia (pGm) affects 20% to 70% of pubertal boys and is usually benign and self-limiting [71,72]. A physical examination may reveal a firm retroareolar mass that may be painful, and the skin is usually intact; however, stretch marks (whitish or reddish) are frequently seen in overweight/obese adolescents, and this is why adolescents seek medical advice [73]. Sometimes, however, the overlying skin might be inflamed in the case of an infection from a juvenile retroareolar cyst, so ultrasound confirmation is warranted.

Ultrasonography can also be useful in differentiating Gm from pseudogynecomastia (pseudo-Gm), the most common cause of Gm, and other rare causes of Gm, such as lipoma, neurofibromatosis, lymphangioma, or hematoma [74,75,76,77,78]. Pseudo-Gm, also called lipomastia, steatomastia, or adipomastia, is caused by fat tissue accumulation resulting in bilateral breast enlargement [74,75,76,77,78].

Considering the high incidence of Gm, further differential diagnoses of its causes should include systemic disorders (e.g., liver disease), iatrogenic Gm in adolescents, especially the consequences of substance use, and endocrine disorders (e.g., adrenocortical cancer, Klinefelter syndrome, partial androgen insensitivity, 11-beta hydroxylase deficiency, or 17-ketosteroid reductase deficiency) [79].

Nowadays, in the obesity epidemic era, patients are presenting with more complex Gm, which is a combination of glandular and fat tissue hypertrophy [80]. From a plastic surgery point of view, the obese male population presents with a new cosmetic problem of breast deformity resulting from the combination of gynecomastia, obesity, and weight loss [81,82].

##### Etiology

Gm results from an imbalance in the estrogen to androgen (E/A) ratio favoring estrogen, as observed also in disorders known to influence E/A balance (Klinefelter syndrome, partial androgen insensitivity syndrome, or aromatase excess syndrome) [75,76,77,78,83]. However, the findings of imbalances in sex hormones in adolescents with Gm are controversial in the literature [74,76,83,84,85,86,87,88]. In a recent, excellent study by Reinehr et al. differentiating pGm from pseudo-Gm with the use of ultrasound evaluation and adjusting the data for testes volumes, pGm was characterized by a relative testosterone deficiency to estradiol concentrations in contrast to pseudo-Gm [74]. The combination of increased serum estrogens in relation to a relatively delayed free testosterone rise and increased tissue sensitivity to normal male levels of estrogen may possibly be a cause of gynecomastia in adolescents [89].

PGm is usually observed at around 13 years of age or at pubertal stage 3 to 4 by the Tanner scale when serum estrogens increase more abruptly than testosterone levels [71,72,78,83]. Limony et al. have shown that pGm appears within a year in relation to the age of peak height velocity (PHV), corresponding to Tanner stage 3 for pubic hair and testicular volumes between 8 and 10 mL [90]. Interestingly, analyzing the GH-IGF-I axis, Reinhert et al.’s group did not find any differences in the IGF-I levels between pGm and pseudo-Gm groups; however, locally produced IGF-1 might be more relevant than circulating IGF-1 for the development of gynecomastia, and hyperinsulinemia in obese adolescents might also potentially influence its occurrence [74,86].

Physiological pGm in adolescents usually resolves within 6 months to 2 years after onset; however, if it persists for over two years or beyond the age of 17 years, further evaluation is suggested [91]. Androgens are aromatized to estrogens in adipose tissues, and as this process is increased in individuals with obesity, including obese pubertal boys, gynecomastia is not only common but persists beyond mid-puberty [72]. As Gm occurs together with pseudo-Gm, breast enlargement can be prominent and affect psychosocial well-being. The primary cause of breast enlargement is the growth of the stroma, accompanied by ductal proliferation and mild Gm during the proliferative phase, which is reversible [72,91,92,93]. However, as presented by Godwin et al., once breast hypertrophy has been present for more than a year, stromal hyalinization and irreversible fibrosis of the parenchyma occur; thus, the only effective treatment at this stage is surgery [80,94]. After massive weight loss (MWL), e.g., after bariatric surgery, the chest may show significant deformities. The surgical treatment of gynecomastia after MWL is complicated due to an excess of skin that sometimes continues in the axilla or dorsal region, a predominantly fatty rather than glandular component, the malposition of the enlarged nipple–areola complex, ptosis, and an inframammary fold that is often marked [82].

##### Psychological Aspects of Gynecomastia and Pseudogynecomastia

Gynecomastia in obese adolescents may have a significant negative influence on their well-being [95]. It can affect social relationships; the behaviors chosen by obese patients can be a source of psychological discomfort associated with negative perceptions by peers but also potentially a concern about sexual identity [96]. Bullying is common and can further aggravate eating disorders, social isolation, and reduced physical activity [92,97].

#### 6.2.2. Functional Hypogonadotropic Hypogonadism in Obese Adolescents

Pediatric and adolescent obesity may influence puberty, as this developmental stage is sensitive to nutrition [98]. It is known from research conducted in obese adult males that obesity can cause suppression of the hypothalamic–pituitary–gonadal (HPG) axis and testosterone secretion [99]. However, in pediatrics, the differential diagnosis of delayed puberty must exclude congenital and acquired/structural causes first. Hypogonadism in adolescents can be caused by gonadal disease (primary hypogonadism), dysfunction of the HPG axis (secondary hypogonadism), be transient/reversible (functional hypogonadotropic hypogonadism, FHH), or be self-limited (constitutional delay in growth and puberty, CDGP). FHH, a transient delay in HPG axis maturation, is usually a consequence of chronic disease, such as celiac disease, inflammatory bowel disease, anorexia nervosa, dysregulated diabetes mellitus, persistent obesity, or iatrogenic impacts (e.g., glucocorticosteroid therapy) [100,101]. CDGP, a nonpathological condition in which the maturation of the HPG axis and PHV are delayed, is the diagnosis for exclusion [102,103]. FHH and CDGP are found most frequently in boys [102,103].

Many research groups focus on the mechanisms underlying the influence of obesity on puberty [104,105,106,107,108,109,110,111,112]. The activation of the HPG axis is triggered by pulsatile GnRH secretion influenced by the activity of KnDY neurons (KNDY: kisspeptin, neurokinin B, and dynorphin) [106,107,108]. Both reproductive KnDY neurons and metabolic neurons (proopiomelanocortin/cocaine, amphetamine-regulated transcript, and agouti-related peptide/neuropeptide Y) interact with GnRH neurons [109,110]. Additionally, the peripheral signaling hormones Y polypeptide, cholecystokinin, insulin, leptin, adiponectin, and ghrelin interact with metabolic neurons related to the HPG axis [109,110,111,112,113,114,115,116,117,118].

There are several studies that evaluate the effect of obesity on the start of puberty in males with controversial results; some studies from Europe and South America associate obesity with early puberty and from North America with a later development of puberty [119,120,121,122,123]. These differences could be due to the way pubertal staging was assessed [104].

Despite different observations on the onset of puberty in obese adolescents, some studies present that, apart from an advanced bone age potentially decreasing final height, persistent obesity may suppress the HPG axis thereafter [104,124].

The incidence of hypogonadism in obese adolescents is not known; however, one study reported a prevalence of hypogonadism (low serum testosterone) of 33% among 14- to 25-year-old males with obesity [125].

There are no pediatric guidelines on the management of FHH secondary to obesity in adolescent males; therefore, FHH should be suspected in obese patients presenting clinical symptoms of hypogonadism, such as delayed or incomplete pubertal development [126].

Obesity and hypogonadism are interconnected; obesity contributes to hypogonadism, and hypogonadism increases obesity [107]. The mechanisms behind hypogonadism in obesity are not well understood. Some researchers propose that obesity causes the suppression of gonadotropins [127,128,129]. Increased peripheral aromatization of testosterone to estradiol in fat tissue contributes to decreased serum testosterone [130,131]. Some authors propose that relative hypogonadism could be related to insulin resistance and imbalances in leptin, sex hormone-binding globulin, growth hormone, and insulin-like growth factor-binding protein-3 ratios [127,128,130,131,132]. Inflammatory proteins such as cytokines and tumor necrosis factor-α, produced in adipose tissues, inhibit kisspeptin secretion, decreasing GnRH secretion [133,134].

Hypogonadism can cause many consequences, from metabolic to psychological [108]. Obese adolescents with pubertal delay are twice as distressed by their conditions, which decreases their quality of life; therefore, this situation warrants detailed evaluation and therapy in Multidisciplinary Endocrine Obesity Centers for Adolescents [82,106]. The European Academy of Andrology (EAA) recommends lifestyle changes as the first line of treatment in patients with functional hypogonadism, since weight reduction can increase testosterone levels as well as improve reproductive function and fertility [135,136,137].

## 7. Summary

We envision that the insights provided in this study will significantly contribute to awareness and understanding of the often-overlooked and non-obvious complications associated with obesity. By shedding light on these intricacies, we aim to empower healthcare professionals with knowledge that can drive more informed decisions and interventions. Obesity, being a multifaceted health concern, necessitates a nuanced understanding of its diverse complications. This work endeavors to unravel its complexities, offering a comprehensive perspective that surpasses surface-level manifestations. This way, we aspire to bridge the gap in knowledge. Moreover, by highlighting non-obvious complications, we aspire to inspire a paradigm shift in how obesity is perceived and managed. Recognizing that certain complications may lurk beneath the surface, undetected for extended periods, underscores the importance of vigilant and comprehensive healthcare practices. Our hope is that this work serves as a catalyst for ongoing research, policy development, and community initiatives aimed at preventing and managing obesity-related complications.

Through increased awareness and a more profound understanding, we can work toward more effective prevention strategies, early interventions, and holistic approaches that address both the visible and concealed challenges posed by obesity.

## Data Availability

Data are contained wihin the article.
